# Chronic kidney disease and kidney health care status: the healthy life in Suriname (HeliSur) study

**DOI:** 10.1007/s11739-018-1962-3

**Published:** 2018-10-26

**Authors:** Rani Nannan Panday, Yentl Haan, Frederieke Diemer, Amar Punwasi, Chantal Rommy, Ingrid Heerenveen, Gert A. van Montfrans, Lizzy M. Brewster

**Affiliations:** 1 Department of Internal Medicine, Academic Medical Center, Amsterdam, The Netherlands; 2 Department of Cardiology, Academic Hospital of Paramaribo, Paramaribo, Suriname; 3 Department of Internal Medicine, Academic Hospital of Paramaribo, Paramaribo, Suriname; 4 Surrenal Dialysis Center, Paramaribo, Suriname; 5 Faculty of Medicine, Anton de Kom University, Paramaribo, Suriname; 6 Nefrosur Medical Centre, Paramaribo, Suriname; 7 Amsterdam Institute for Global Health and Development, AHTC, Tower C4, Paasheuvelweg 25, BP 1105 Amsterdam, The Netherlands; 8 Creatine Kinase Foundation, Amsterdam, The Netherlands

**Keywords:** Cardiovascular risk factors, Chronic kidney disease, Renal replacement therapy, Low- and middle-income countries, Kidney health care

## Abstract

The high cardiovascular risk burden in low- and middle-income countries is expected to lead to an explosive increase in chronic kidney disease (CKD). However, population data on CKD from these countries are scarce. Therefore, we assessed kidney health in Suriname. In the Healthy Life in Suriname (HeliSur) study, a random sample of the adult population, we collected data with standardized questionnaires, physical examination, and blood and urine samples analysed in a central laboratory. Prevalent CKD was graded with KDIGO guidelines. In addition, we assessed national data on prevalent renal replacement therapy (RRT), estimated the future need for RRT, and evaluated national kidney health work force and policies. We include 1117 participants (2.0‰ of the population), 63% women, 40% of African ancestry and 43% of Asian ancestry, with a mean age of 42.2 (SE 0.4) years. Blood pressure is elevated in 72% of the participants, 26% have diabetes or prediabetes, and 78% are obese or overweight. The prevalence of CKD is 5.4%, and around 0.3% have kidney failure, translating to approximately 1500 patients nationally (2690 per million population, pmp), with currently 516 patients (920 pmp) on dialysis. Based on the participants from the random population sample in CKD stage G3 or G4, we estimate that 6750–10,750 pmp may develop kidney failure within the next 10 years. However, specialized kidney health workforce is currently very limited, and specific national or local policies for CKD management are lacking. Since the large majority of the general population has one or more risk factors for CKD including elevated blood pressure, urgent action is needed to strengthen kidney health care and prevent a catastrophic rise in need for RRT in the coming years.

## Introduction

Chronic kidney disease (CKD) is increasingly recognized as a major cause of premature death [1]. The rising prevalence in CKD is driven by the global increase in the prevalence of diabetes mellitus, hypertension, and obesity, which disproportionately has an impact upon low- and middle-income countries [1–6].

However, the absence of CKD registries in most of the low- and middle-income countries renders it difficult to ascertain the true burden of kidney disease in these countries. In this study, we assess the kidney health care status in Suriname, a middle-income country in South America.

## Methods

### Study area and design

Suriname is a Caribbean nation with approximately 560,000 citizens who are mainly of African or Asian (South Asian, Indonesian, and Chinese) ancestry. More than 70% of the population lives in an urban setting [7–12]. The gross national income per capita (i.e. the gross national income converted to US dollars using the World Bank Atlas method divided by the midyear population) is around 8000 dollars (2015) [8]. Literacy rate, persons of age 15 and over who can read and write in Dutch, the official language, is 95.6% of the population, men 96.1% and women 95% (2015) [9]. Total health expenditure (2015) is 6.5% of the gross domestic product [8], and life expectancy at birth is around 72 years (2015) [8–11]. A combination of public and private health care systems funded by national taxation and income contributions provide primary through tertiary medical care and essential medicines for the large majority of the population including the poor and near poor, without substantial consumer co-payment at the point of care delivery [10–12].

Diseases of the circulatory system are the main cause of death [7, 10]. The Healthy Life in Suriname (HeliSur) study was the first major scientific study, conducted from 2014 to 2017 in a random sample of the urban population, designed to assess cardiovascular risk factors as well as asymptomatic and symptomatic target organ damage including CKD. Study procedures and selected cardiovascular parameters from the HeliSur study were reported previously [7]. In this study, we focus on CKD and its predictors, and estimate the current and future dialysis need within the context of national kidney health care.

In brief, we chose a random population sample of 1800 men and women aged 18–70 years to be interviewed at home. All participants gave written informed consent to participate. The interview included demographic, socioeconomic, dietary, and health-related questions. Subsequently, participants were invited for physical examination, and for laboratory blood and urine tests in the local hospital administered by trained medical staff. The study was conducted according to the principles of the Declaration of Helsinki (59th WMA General Assembly, Seoul, October 2008) and in accordance with the Medical Research Involving Human Participants Act. Ethical clearance was obtained from the Ethics Committee of the Ministry of Health in Suriname in 2012 (VG021-2012). In addition, we evaluate the national kidney health care status, including “prevalent” renal replacement therapy (RRT), RRT gap, the health work force, and national policies (2016–2017).

### Outcomes

The primary outcome is the national prevalence of CKD. The secondary outcome is the prevalence of RRT. Further outcomes are determinants of CKD and glomerular filtration rate (GFR); characteristics of participants with proteinuria; the current status of kidney health care including the number of nephrologists and specialized nurses, RRT capacity and RRT gap, and evaluation of national or regional programs or policies on early detection and treatment of CKD.

### Data collection

To assess the cardiovascular profile and CKD in the random population sample, history taking was by trained and qualified staff, under supervision of a board certified cardiologist. Height and weight were measured twice to the nearest 0.1 cm and 0.1 kg. Body mass index (BMI) was computed as mean weight (in kilograms) divided by the mean height (in metres) squared. Blood pressure was measured twice in the sitting position with an automated oscillometric device (WatchBP Office; Microlife AG, Widnau, Switzerland), and an appropriately adjusted cuff size on the left upper arm supported at heart level. The ankle–brachial index was assessed twice in a supine position by WatchBP Office ABI (Microlife A.G., Widnau, Switzerland). A 12-lead electrocardiogram was recorded with the ECG-1200 Biocare (Collateral Medical, Mumbai, India).

We assessed pulsatile arterial haemodynamics including pulse wave velocity twice with the Arteriograph (TensioMed, Budapest, Hungary). Fasting venous blood samples were collected for laboratory measurements at the Academic Hospital of Paramaribo, Suriname. Plasma creatinine was estimated with the compensated Jaffé alkaline picrate colorimetric creatinine assay (UniCel DxC 600/800 System Beckman Coulter, Brea, California, United States) [13, 14]. As recommended by the Kidney Disease Improving Global Outcomes (KDIGO) guidelines, we collected early morning spot urine samples [15, 16]. Proteinuria was assessed with a reagent strip with automated reading (Uriscan Strip, Uriscan Super YD Diagnostics Yongin-Si, Kyunggi-Do, South Korea).

To assess the number of patients per million population (pmp) receiving dialysis care, we sought data from all dialysis centers through personal contact and collected data from the National Statistics Suriname. Using projections on population growth, we estimated the future prevalence of kidney failure, mainly based on the number of patients currently in CKD stage G3 [17, 18].

Finally, we collected data from health workers and policy makers, published scientific literature, government reports, and other relevant data sources including grey literature or reports with limited circulation where available, on national CKD health staff and health policies, and on RRT capacity, and we assessed whether there are active CKD prevention, detection, or referral and management programs or guidelines [6]. Because of the very small numbers of specialized health workers expected in kidney care, we did not carry out a pilot for this part of the study [19], but took a pragmatic, explorative approach in selecting questions with local relevance from the International Society of Nephrology survey [6].

### Chronic kidney disease categories

CKD, the main outcome, was diagnosed according to the recommendation of the KDIGO guidelines based on proteinuria and estimated glomerular filtration rate (eGFR) expressed in mL/min/1.73 m^2^ [16]. In accordance with the KDIGO guidelines, five eGFR categories (G1, eGFR ≥ 90; G2, 60–89; G3a, 45–59; G3b, 30–44; G4, 15–29, and G5, < 15 mL/min/1.73 m^2^) were used. GFR was estimated with “the Chronic Kidney Disease Epidemiology Collaboration” (CKD-EPI) equation for values > 60 mL/min/1.73 m^2^, as this method offers an improved reproducibility and accuracy at higher GFR levels. We used the Modification of Diet in Renal Disease (MDRD) equation for GFR ≤ 60 mL/min/1.73 m^2^.

Albuminuria categories were estimated with a protein reagent strip: A1, negative, A2, trace (100–300 mg/L), and A3,  ≥ 1 + or ≥ 300 mg/L, using automated machine read outs [20, 21]. Because of the small numbers expected, A2 and A3 were taken together in the analyses. CKD was defined as an eGFR < 60 mL/min/1.73 m^2^ or the presence of proteinuria (stage A2/A3). Kidney failure was defined as CKD stage G5 (eGFR < 15 mL/min/1.73 m^2^) or on RRT [16]. Participants with missing parameters to estimate CKD were excluded. The current and future need for maintenance dialysis were estimated based on the prevalence of CKD stage G3, assuming that 25% of stage G3A and 50% of G3B patients progress to stage G5 in 10 years [17, 18], and that half of these will be on maintenance dialysis [22]. In different scenarios, patients in stage G4 were assumed to develop 100%, 50%, or 0% (because of mortality) kidney failure in 10 years [23, 24].

### Other definitions

Ancestry was self-defined. Hypertension was defined as systolic blood pressure ≥ 140 mmHg, or diastolic blood pressure ≥ 90 mmHg, or receiving antihypertensive drug therapy; and prehypertension as systolic blood pressure 120–139 mmHg or diastolic blood pressure 80–89 mmHg, without antihypertensive drug therapy. Controlled hypertension included subjects with hypertension using antihypertensive medication with a systolic blood pressure < 140 mmHg and diastolic blood pressure < 90 mmHg.

We used ancestry-specific cut-off values for BMI: obesity ≥ 30 kg/m^2^ in African Surinamese participants (i.e. Creole and Maroons) and ≥ 27.5 kg/m^2^ in Asian-Surinamese participants (i.e. Surinamese of South Asian and other Asian ancestry). Overweight was defined as body mass index 25.0–29.9 kg/m^2^ and 23.0–27.4 kg/m^2^, respectively, in African and Asian ancestry participants [7].

We considered participants to be diabetic if glucose was 7.0 mmol/L or higher or when glucose-lowering medication was used, while prediabetes was defined as glucose 5.6–6.9 mmol/L without the use of glucose-lowering drugs. Diabetes control was defined as glucose lower than 7.0 mmol/L on treatment.

Dyslipidaemia was defined as having at least one of the following: total cholesterol ≥ 6.2 mmol/L, low-density lipoprotein cholesterol ≥ 4.1 mmol/L, high-density lipoprotein cholesterol < 1.0 mmol/L, triglycerides ≥ 2.3 mmol/L, or the use of lipid-lowering drugs, with control defined as reaching non-dyslipidaemic levels with therapy. Borderline dyslipidaemia was defined as total cholesterol 5.2–6.1 mmol/L, low-density lipoprotein cholesterol 3.4–4.0 mmol/L, high-density lipoprotein cholesterol 1.0–1.6 mmol/L, or triglycerides 1.7–2.2 mmol/L without the use of lipid-lowering drugs.

Asymptomatic organ damage was defined based on the European Society of Hypertension/European Society of Cardiology guidelines [25], as the presence of pulse pressure ≥ 60 mmHg; or left ventricular hypertrophy, Sokolow−Lyon criteria: S in V1 or V2 + R in V5 or V6 (whichever is larger) ≥ 35 mm, or R in aVL ≥ 11 mm; or carotid–femoral pulse wave velocity > 10 m/s; or ankle–brachial index < 0.9; or CKD stage G3, eGFR 30–59 mL/min/1.73 m^2^; or stage A2 proteinuria (trace with the dipstick in spot urine), without established cardiovascular disease present.

Established cardiovascular or renal disease was defined as any of the following, a history of cerebrovascular disease including ischemic stroke, cerebral haemorrhage, or transient ischemic attack; coronary heart disease including myocardial infarction, angina, or myocardial revascularization; heart failure; symptomatic lower extremities peripheral artery disease; CKD stage G4 to G5, eGFR < 30 mL/min/1.73 m^2^; stage A3 proteinuria (≥ 1 + with the dipstick in spot urine).

Finally, educational level was assessed through the highest completed level of education, with low education level defined as having primary education (UNESCO International Standard Classification of Education level 1), or less [7].

### Statistical analyses

Appropriate statistical tests were used depending on type and distribution of data. Specifically, we calculated descriptive data including CKD by age categories (below 50 years vs 50 years or older) and gender, and computed correlates of eGFR and CKD, including age, gender, ancestry, BMI, blood pressure, and glucose with the two-tailed Pearson product−moment correlation coefficient between ranked variables (Spearman’s rho) before entering relevant variables into multivariable regression analysis, using forced entry. We selected established risk factors as predictors including age, gender, ancestry, and blood pressure and glucose parameters, as well as other variables significant at *p* < 0.05. We chose continuous variables as the primary analysis, and dichotomous variables for blood pressure and diabetes mellitus as the secondary analysis. Gender and ancestry were predefined subgroups. Missing data were not imputed. Because of the small sample size expected for proteinuria, we only report descriptive variables and frequencies. In sensitivity analyses, we reanalysed the predictors of eGFR and CKD by ancestry; excluded persons with underweight (BMI < 18.5 kg/m^2^) because of potential low creatinine with overestimation of the kidney function [26]; and reanalysed the data estimating GFR ≤ 60 mL/min/1.73 m^2^ with the CKD-EPI equation. Outcomes in parentheses are mean (SE) and in square brackets are 95% confidence intervals unless stated otherwise. *p* values are two-tailed unless stated otherwise. We did not adjust *p* values for multiple outcomes, but limited formal statistical testing on non-primary outcomes, and only used one-sided *p* values with pre-existent evidence or a hypothesis on the direction of the outcome. Data were analysed using SPSS statistical software package for Windows, version 24.0 (SPSS Inc., Chicago, IL, USA).

## Results

### Random population sample

We had data available of 1159 participants, a response rate of 78% [7]. For this analysis, a total of 42 participants were excluded, 35 with missing creatinine and a negative dipstick test, 4 with normal eGFR and missing data on urine protein, and 3 with missing data on creatinine and urine protein. There are no significant differences in the presence of hypertension or diabetes mellitus in participants with or without missing CKD component data, respectively, 38.1 and 12.8% in the excluded, vs 40.4 and 14.6% in the included participants (*n* = 1117).

Selected baseline characteristics of the participants are depicted in Table 1. The mean age is 42.2 (0.4) years, and 40.1% are of African and 43.1% of Asian ancestry. The high cardiovascular risk is striking, with 72% being prehypertensive or hypertensive, 26% with diabetes or prediabetes, 78% are overweight or obese, 30% ever smoked, and 96% have dyslipidaemia or borderline dyslipidaemia, which is mainly driven by borderline HDL in 74% of the participants.

**Table 1:** Cardiovascular health in the random population sample by gender

Parameters	All (*N* = 1117)	Men (*n* = 417)	Women (*n* = 700)
Age, years	42.2 (0.4)	42.7 (0.7)	42.0 (0.5)
Ancestry, African/Asian^a^	40.1/43.1	38.6/43.4	41.0/42.9
Tobacco use	30.3	55.4	15.4
Low education level	38.0	32.9	41.0
Body mass index, kg/m^2^	27.8 (0.2)	25.6 (0.2)	29.1 (0.2)
Overweight	41.2	45.1	38.9
Obesity	36.6	20.6	46.1
(pre)HT and/or (pre)DM	74.6	81.8	70.2
Systolic BP, mmHg	129.7 (0.6)	131.4 (0.9)	128.8 (0.8)
Diastolic BP, mmHg	81.3 (0.3)	83.5 (0.6)	79.9 (0.4)
Prehypertension	31.3	36.4	27.2
Hypertension	40.4	41.4	39.8
HT controlled^b^	20.9	15.7	24.2
Prediabetes mellitus	11.7	11.8	11.6
Diabetes mellitus	14.6	13.2	15.5
DM controlled^b^	20.9	14.5	24.1
Cholesterol, mmol/L	4.7 (0.0)	4.6 (0.0)	4.8 (0.0)
Borderline dyslipidaemia	54.9	49.6	59.3
Dyslipidaemia	41.3	49.6	36.3
DL controlled^b^	3.0	2.9	3.2
Proteinuria	2.9	3.8	2.3
CKD	5.4	6.5	4.7
Asymptomatic TOD	23.1	21.6	24.0
Cardiovascular disease	15.0	14.9	15.0

Point estimates with data in brackets are means (SE); other data are percentages unless indicated otherwise. Tobacco use is defined as ever smoked; please see the Methods for other definitions

*CKD* chronic kidney disease, *TOD* target organ damage

^a^The remaining participants were of other ancestry

^b^HT (DM, DL) controlled, the percentage of participants with adequately treated hypertension (diabetes, dyslipidaemia) as a percentage of all persons with the condition

### Chronic kidney disease

Tables 1, 2, 3 and 4 depict proteinuria, eGFR, and CKD as found in the random population sample. The number of persons with proteinuria is small, but the data indicate that the prevalence is higher in relatively young, African ancestry men with a high and uncontrolled risk factor burden, and organ damage outside the kidney (Table 2).

**Table 2:** Cardiovascular health in participants with CKD and with proteinuria

Parameters	CKD (*n* = 60)^c^	Proteinuria (*n* = 32)
Age, years	49.4 (2.0)	40.3 (2.3)
Men	45.0	50.0
Ancestry, African/Asian^a^	35.0/45.0	46.9/31.3
Tobacco use	38.3	37.5
Low education level	41.7	34.4
Body mass index, kg/m^2^	27.7 (0.8)	27.5 (1.2)
Obesity	45.0	37.5
Systolic BP, mmHg	138.1 (3.0)	140.8 (4.1)
Diastolic BP, mmHg	83.4 (1.7)	86.8 (2.5)
Prehypertension	16.7	28.1
Hypertension	66.7	50.0
HT Controlled^b^	28.2	0.0
Prediabetes mellitus	13.3	9.4
Diabetes mellitus	30.0	21.9
DM Controlled^b^	11.1	14.3
Dyslipidaemia	48.3	53.1
eGFR, mL/min/1.73 m^2^	71.1 (4.7)	93.3 (6.3)
CKD G3 or higher	55.9	16.1
Asymptomatic TOD	60.0	62.5
aTOD excl. CKD	33.3	31.3
Cardiovascular disease	40.0	37.5
CVD excl. CKD	23.3	12.5

Point estimates with data in brackets are means (SE); other data are percentages unless indicated otherwise. Tobacco use is defined as ever smoked; please see the Methods for other definitions

*eGFR* estimated glomerular filtration rate, *CKD* (*G3*) chronic kidney disease (stage G3), *TOD *(asymptomatic) target organ damage, *CVD* cardiovascular disease

^a^The remaining participants were of other ancestry

^b^HT (DM) controlled, the percentage of participants with adequately treated hypertension (diabetes) as a percentage of all persons with the condition

^c^Including one patient with eGFR 6 mL/min/1.73 m^2^ without urine sample or proteinuria status

The mean population eGFR is 107.6 (0.7) mL/min/1.73 m^2^. The estimated GFR is lower in men and in participants of Asian ancestry, respectively, 96.2 (1.5) vs 109.6 (1.9) mL/min/1.73 m^2^ in Asian vs African ancestry men, and 106.5 (1.2) vs 117.9 (1.4) in women. Furthermore, eGFR is negatively correlated with age, BMI, blood pressure, glucose, and cholesterol, but not with education level. In multivariable regression analysis, Asian ancestry and male gender are the main (negative) predictors of eGFR (Table 3).

**Table 3:** Predictors of eGFR in the general population

Variable	Correlation coefficient^a^	Regression coefficient	Confidence interval (95%)	
			Lower bound	Upper bound
Intercept		163.57	152.69	174.45
Age	− 0.43	− 0.71	− 0.82	− 0.59
Asian ancestry	− 0.28	− 10.80	− 13.49	− 8.10
Men	− 0.24	− 9.40	− 12.16	− 6.63
SBP	− 0.28	− 0.07	− 0.14	0.01
Glucose	− 0.21	0.06	− 0.56	0.67
Cholesterol	− 0.17	0.84	− 0.46	2.15
BMI	− 0.06	− 0.09	− 0.32	0.14

Univariable and multivariable analyses of predictors of the estimated glomerular filtration rate (eGFR) (mL/min/1.73 m^2^) in the general population

^a^Two-tailed Spearman’s rho (*p *< 0.001, except for BMI, *p *< 0.05)

Around 5.4% of the population have CKD (Table 4); 3.3 vs 9.5% in participants < 50 vs ≥ 50 years; and 8.9% vs 3.6% in those with vs without target organ damage. CKD prevalence in Asian vs African ancestry men, respectively, is 7.2 vs 6.8%, with 4.7 vs 3.5% in women. CKD is significantly correlated with (rho), age (0.12), systolic blood pressure (SBP) (0.09), and glucose levels (0.08) (*p *< 0.01), but not with ancestry; with marginal significance of these predictors in logistic regression analysis, OR for glucose 1.15 [1.05–1.27], age 1.02 [1.00–1.05], and SBP 1.02 [1.00–1.03].

**Table 4:** Chronic kidney disease

CKD	Percentage of the population by eGFR and albuminuria category: KDIGO and HeliSur study			Albuminuria stages		All
	Grade	Function	eGFR	A1	A2 and A3	
eGFR stages, description and range (mL/min/1.73 m^2^)	G1	Optimal	≥ 90	78.2	1.5	79.7
	G2	Mildly decreased	60–89	16.5	0.8	17.3
	G3a	Mildly to moderately decreased	45–59	1.7	0.2	1.9
	G3b	Moderately to severely decreased	30–44	0.4	0.1	0.4
	G4	Severely decreased	15–29	0.3	0.1	0.4
	G5	Kidney failure	< 15 or RRT	0.1	0.1	0.3^a^
	All			97.0	2.9^b^	100^a^

Chronic kidney disease (CKD) by estimated glomerular filtration rate (eGFR) and albuminuria category based on Kidney Disease: Improving Global Outcomes (KDIGO) guidelines

*RRT* renal replacement therapy

5.6% had CKD, including:

^a^One participant with kidney failure and anuria without proteinuria classification

^b^One participant with proteinuria and missing creatinine. Values in cells are percentages, and may not add up because of rounding

Since patients with treated hypertension and diabetes might have experienced organ damage not reflected in current blood pressure and glucose values, we replaced blood pressure and glucose as continuous variables, and entered hypertension and diabetes mellitus as dichotomous predictors in the multivariable regression analyses. Hypertension (but not diabetes mellitus) is a significant predictor of eGFR with a beta of  − 4.20 [− 7.25 to −1.15], and became the only significant predictor for CKD, with an odds ratio of 3.91 [1.79–8.51].

Forty-six participants have a BMI < 18.5 kg/m^2^, including two with CKD, one with diabetes and a plasma creatinine of 350 μmol/L without proteinuria, and one with proteinuria. Reanalysing the data excluding these participants; predicting eGFR and CKD by ancestry; or estimating GFR ≤ 60 mL/min/1.73 m^2^ with the CKD-EPI equation does not change the magnitude or the direction of the outcomes.

### Renal replacement therapy

#### Dialysis service delivery

Patients are dialyzed in six national dialysis clinics (2016), one state-owned National Dialysis Center and five private clinics, providing a capacity for maintenance (haemo-) dialysis of around 500 patients (900 pmp). All treatments are covered by the health insurance, and patients need no payment or co-payment at the point of care. The National Dialysis Center serves around 30–50% of all dialysis patients. The National Dialysis Center also collects national data from private clinics. Peritoneal dialysis and kidney transplantation were not offered in Suriname at the time of this survey.

#### Current RRT

**Fig. 1:**
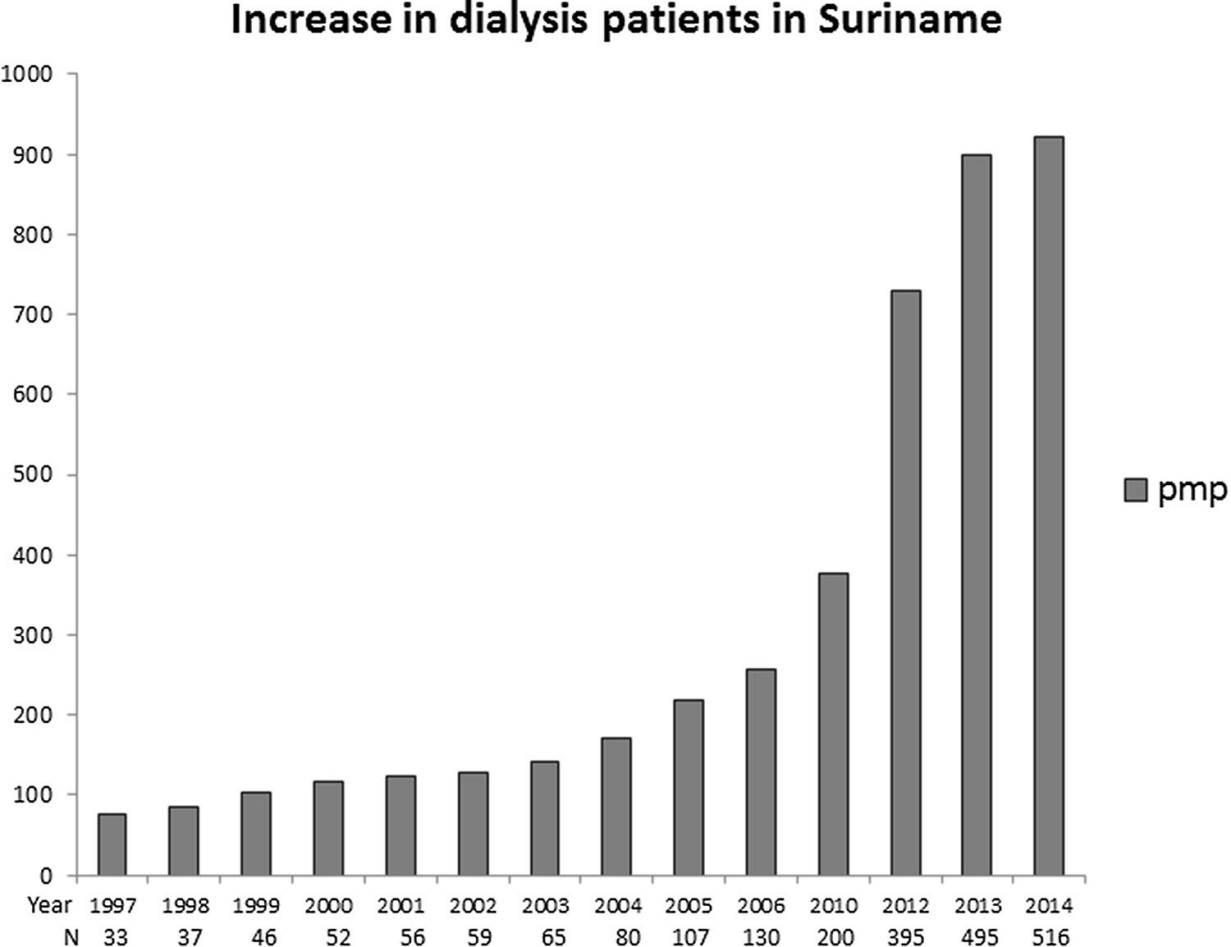
Rapid increase in reported dialysis patients in Suriname. The national registry included all (haemo-) dialysis patients in an increasing number of state-owned and private clinics, from 1997 to 2014. No data are available for 2007, 2008, 2009 and 2011. *PMP* patients per million population, *N* total number of patients

According to our survey in 2016 at the private clinics and the National Dialysis Center, 516 unique patients are on acute or maintenance dialysis care as for 2014, which is a large increase compared to the years before (Fig. 1). There are no data available on acute vs maintenance dialysis, but according to local health workers, the large majority of these patients, around 80%, are on maintenance dialysis.

#### RRT need

Based on the population size of Suriname of around 560,000 residents at the time of the study [27], and the current rate of patients with kidney failure (0.3%, Table 4), we estimate that approximately 1500 patients (2690 pmp) have kidney failure (calculated from unrounded data). With around 516 unique patients with acute or chronic kidney failure (920 pmp) on dialysis in 2014, according to the ad hoc interviews with local kidney health care staff (*n* = 5), there is a maintenance dialysis care gap, and linkage to care of patients with CKD may be insufficient. Patients may die, mainly of CVD, without CKD being diagnosed. Furthermore, health workers mentioned that patients also refuse dialysis treatment, or may lack the financial means for the out of pocket expense to travel to the clinic.

#### Future RRT

Based on the participants with CKD stage G3/G4 at the time of the study (2.7%), we conservatively estimated the number of patients with kidney failure to accrue to 4175–6650 (6750–10,750 pmp) in 10 years, assuming a population growth of 1% per year [27], and an unchanged distribution of kidney function stages and age. This projected four- to sixfold increase in kidney failure is a rather conservative estimate, based on disease progression in persons of European ancestry receiving intensive treatment [17, 18]. When taking into account the faster progression to kidney failure in patients of South Asian and African ancestry [18], the very high risk factor burden in the population, and the limited predialysis care in this country, it becomes clear that these factors will potentially create a disastrously large increment in dialysis need (or premature death) in the coming years.

#### CKD care organization

There are three Surinamese nephrologists (around five pmp) for whom training abroad was mandatory, and 20 Surinamese specialized dialysis nurses (36 pmp), who are locally trained. Additionally, there is a variable number of specialized nurses from abroad (mainly the Philippines) present. There is no active national nephrology society, and no organized system or structure to identify persons with risk factors for CKD, including none for diabetes and hypertension. In addition, there is no national or local CKD detection program based on policy or guidelines. Persons with diabetes and hypertension who are in care are screened on an individual basis by their physician with plasma creatinine estimations, but eGFR or CKD stages are not routinely assessed. There is no national or local health policy on CKD, no standard care plan, no organized system to ensure that persons with established CKD receive guideline-concordant clinical care, whereas no national CKD guideline has been formulated. Furthermore, the country has no CKD registry, and CKD is not recognized to be a health priority by the Surinamese government. There is no CKD advocacy group at the higher levels of government, or non-governmental organization to raise the profile of CKD and its prevention, and there are no existing national/regional physician oriented organizations or patient organizations that provide resources for CKD prevention, detection, or management. Finally, in the absence of national funding opportunities, research capacity on kidney disorders is very limited, and we retrieved no previously published scientific report or national report on CKD in Suriname.

## Discussion

In this study, we find that within the context of a very high cardiovascular risk burden, with around 75% of the population having (pre)diabetes or (pre)hypertension, 5.4% have CKD, and 0.3% (around 2690 pmp) have end-stage renal disease. A total of 516 patients (920 pmp) receive haemodialysis, a sharp rise compared to previous years. Although the rise in haemodialysis is a worldwide trend [28], the nearly tenfold increase in about 15 years is striking. Moreover, we conservatively estimate based on the number of patients currently with CKD, that CKD stage G5 (kidney failure), may rise to 6750–10,750 pmp in 10 years, a growth rate far in excess of the estimated population growth [27]. This potential catastrophic increase that also outpaces global projections [28] is highly likely to be devastating to patients, the work force, and the health care system of this middle-income country.

Compared to European countries, CKD prevalence in this country is on the lower end of the spectrum at 5%, with most countries having CKD rate around 10%, while dialysis rates are relatively high, compared to less than 500 pmp in most developed countries [29, 30]. Suboptimal CKD diagnosis, the absence of other treatment options, including predialysis care and renal transplantation, or faster progression to dialysis because of the high and largely uncontrolled cardiovascular risk burden in persons of African and Asian ancestry might explain this discrepancy between the relatively low CKD rate vs high dialysis rate.

CKD and RRT prevalence data from other countries in the Caribbean region are scarce, but hypertension and diabetes mellitus are recognized to be the leading causes of CKD and kidney failure in the region [31]. In French Guyana, the reported RRT prevalence for 2011 is 1553 pmp [32]. Thus, the efforts of the Strategic Plan of the Pan American Health Organization 2014–2019, directed towards achieving RRT capacity of at least 700 patients per million population by 2019 [33] will probably be insufficient for Caribbean countries.

The status of kidney health care as evaluated by this study indicates that the health system of Suriname faces substantial challenges to meet the current and future health needs of persons with CKD. The number of nephrologists and specialized nurses is low [30], and specific policies for CKD prevention and management, or CKD surveillance are lacking. Although citizens are insured for health care costs without point of care co-payment, health care workers have mentioned patient’s barriers to access care, including costs of transport to the clinic, loss of income when visiting the clinic, or cultural belief systems surrounding CKD and dialysis care, which should be further explored.

Importantly, as reported by the Inter-American Development Bank, there is limited availability of funding for health, including CKD care (RRT technologies, essential medicines, service delivery and infrastructures, and kidney disease detection), and reforms are needed to ensure financial sustainability of the health sector in this country [11]. Notably, in Suriname, there is a higher degree of separation between financing and provision of services than other countries in the region. Health insurance institutions are still exclusively payers and not also health service providers as in many countries in the region. Citizens expect high quality of care and access to technology and high cost procedures. These forces increase costs, and may make it difficult to introduce reforms that reduce the fees for doctors and clinics, the package of benefits for consumers, or increase user’s payments; measures needed to create a more efficient and sustainable health sector payments system [11].

Within this context, investments in health promotion and prevention to reduce CKD become urgent. Health policies should focus on reduction of the cardiovascular risk burden, CKD, and the progression to end organ damage including kidney failure, to help reduce the overwhelmingly high medical and economic burden of morbidity and mortality resulting from CKD in this middle-income country setting.

This study has several strengths and limitations. A main strength is that we provide data on the kidney health care status of a country in the Caribbean region, where national and regional data on CKD and kidney health care systems are limited or absent. We carefully assessed the complete kidney health status, including cardiovascular risk burden, symptomatic and asymptomatic end organ damage, CKD prevalence based on a random population sample, national dialysis data, and assessments of specialized staff and CKD health care policies. The population sample is sizable at around *N* = 1100 with a population size of 560.000 (2.0 participants per 1000 population members), two orders of magnitude larger than for example the US NHANES study, with 0.05 participants per 1000 population members [7].

However, we worked in resource-restrained settings. We assessed proteinuria in the random population sample with a dipstick with automated (machine) reading, which is widely used in low- and middle-income settings, but may underestimate proteinuria [20, 21]. In addition, creatinine and proteinuria were measured only once in this cross-sectional setting. Furthermore, analytical performance and specificity of the plasma creatinine assay are critical determinants of the eGFR accuracy [16]. We assessed plasma creatinine with the compensated Jaffé alkaline picrate colorimetric creatinine assay, which might be affected by pseudo-creatinine chromogens (glucose, proteins, ketone bodies), but studies using both analytical and biological variability criteria reveal a low risk (4%) for misclassification of CKD based on Jaffé assay results in diabetics and nondiabetics, mainly occurring around 60 mL/min/1.73 m^2^, where we used MDRD equation to optimize the classification. Thus, the widely used compensated Jaffé creatinine procedure, in spite of the glucose-dependent bias, is considered a reliable and cost-effective tool for the renal function assessment [13, 14].

Our study is further limited by suboptimal disease registries, and data on gender or age of patients on RRT are not available. However, we do report outcomes by gender for cardiovascular risk factors and CKD in the random population sample, where our evidence indicates that men are less inclined to participate [7], but have a higher burden of (uncontrolled) cardiovascular risk factors, cardiovascular disease, proteinuria, and CKD.

The lack of infrastructure to perform renal biopsies and lack of data on kidney disease with normal GFR might have led to an underestimation of the true prevalence of CKD. However, gross underreporting of patients on dialysis is unlikely, because of the relatively small population size and number of dialysis clinics, and the state-funded reimbursement per dialysis patient in care. The health care workers perceive a gap in linkage to care of patients in need of dialysis, which might be related to the lack of CKD detection and management policies, patients visiting dialysis clinics in neighbouring countries [32], or active avoidance of dialysis care. We did not address this gap or the perceptions of the citizens regarding CKD and dialysis. Finally, we did not collect data on acute kidney injury, on citizens’ barriers to access kidney care, or on outcomes for CKD patients and patients on RRT.

In summary, chronic non-communicable diseases including CKD continue to be a major public health concern in low- and middle-income countries. Patients with CKD have high rates of healthcare utilization, morbidity, and mortality [34], and hence constitute a significant economic and clinical burden to the healthcare system of these nations. We provide data in a Caribbean setting, where against a back drop of a very high cardiovascular risk factor burden, CKD prevalence is around 5% with a relatively high prevalence of kidney failure of around 2690 pmp, and currently a dialysis rate of around 920 pmp. Without preventive measures, a catastrophic increase in maintenance dialysis need is expected, with kidney failure rates up to 10,000 pmp within 10 years. With a low number of specialized kidney health care staff currently providing maintenance dialysis, the health care system seems ill prepared to handle such a sharp increase in demand for kidney health care. As CKD outcomes not only include progression to kidney failure, but also an increased risk of cardiac events and stroke [34], urgent action is needed. Currently, the country has limited capacity and readiness to provide this level of CKD care. This Caribbean nation should clearly set the agenda to drastically reduce cardiovascular risk burden, actively screen the population at risk for CKD, and develop a strong incentive to adapt the health care system to provide CKD management and predialysis care.
